# Apologetic

**Published:** 1886-01

**Authors:** 


### APOLOGETIC.

We are obliged to ask forbearance from our correspondents. There are articles from valued contributors for which we cannot at present find room. They will be inserted all in good time, but space is limited, and we cannot get one hundred pages of reading matter into a fifty-six page journal. We are obliged to omit the letter to Junior Dentists, as well as other editorial articles. Judging from the many letters of commendation which we have received, this will be a disappointment to some, but the subject will be resumed next month. We have changed the type and the make-up of "Current News" that we may gain a little space, but even this does not relieve the pressure. We feel under obligations to those who have favored the journal with articles, and can assure them that all such as have been accepted will see the light as soon as possible.

